# Evaluation of the effect of tofogliflozin on the tissue characteristics of the carotid wall—a sub-analysis of the UTOPIA trial

**DOI:** 10.1186/s12933-022-01451-6

**Published:** 2022-02-05

**Authors:** Naoto Katakami, Tomoya Mita, Norikazu Maeda, Yasunori Sato, Hirotaka Watada, Iichiro Shimomura, The UTOPIA Study Investigators

**Affiliations:** 1 Department of Metabolic Medicine, Osaka University Graduate School of Medicine, 2-2, Yamadaoka, Suita, Osaka 565-0871 Japan; 2 Department of Metabolism & Endocrinology, Juntendo University Graduate School of Medicine, Hongo 2-1-1, Bunkyo-ku, Tokyo, 113-8421 Japan; 3 Department of Metabolism and Atherosclerosis, Osaka University Graduate School of Medicine, 2-2, Yamadaoka, Suita, Osaka 565-0871 Japan; 4 Department of Preventive Medicine and Public Health, Keio University School of Medicine, 45 Shinanomachi Shinjuku-ku, Tokyo, 160-8582 Japan

**Keywords:** Atherosclerosis, Diabetes, Carotid artery, Tissue characteristics, SGLT2 inhibitor, Tofogliflozin

## Abstract

**Background:**

Since sodium-glucose cotransporter 2 (SGLT2) inhibitors have a pleiotropic antiatherogenic effect, they are expected to attenuate the progression of atherosclerosis. However, whether SGLT2 inhibitors affect the tissue characteristics of the human arterial wall remains unclear. This study aimed to evaluate the effects of tofogliflozin, a selective SGLT2 inhibitor, on the tissue characteristics of the human arterial wall in type 2 diabetes (T2DM) patients without apparent cardiovascular disease (CVD).

**Methods:**

The present study was a post hoc analysis based on data obtained from the Using Tofogliflozin for Possible Better Intervention against Atherosclerosis for Type 2 Diabetes Patients (UTOPIA) trial, which was a multicenter prospective, randomized, open-label, blinded-endpoint study conducted to evaluate the efficacy of tofogliflozin in preventing the progression of atherosclerosis in patients with T2DM. We evaluated the longitudinal change in the ultrasonic tissue characteristics of the carotid wall using gray-scale median (GSM), an established index of ultrasonic tissue characteristics. The right and left intima-medial areas were delineated, and the GSM values were evaluated (right GSM-CCA and left GSM-CCA). The average values of the right and left carotid arteries were defined as “mean GSM-CCA value.”

**Results:**

In a mixed-effects model for repeated measures, mean GSM-CCA, along with the right and left GSM-CCA values, did not significantly change in either the tofogliflozin (n = 168) or conventional treatment group (n = 169). In addition, the tofogliflozin and conventional treatment groups did not significantly differ regarding the change of the mean GSM-CCA (mean difference [95% CI] − 1.24[− 3.87, 1.38], P = 0.35), along with the right (mean difference [95% CI] − 2.33[− 5.70, 1.05], P = 0.18) and the left GSM-CCA (mean difference [95% CI] − 0.29 [− 3.53, 2.95], P = 0.86) values. Similar findings were obtained even after adjusting for traditional cardiovascular risk factors and/or the administration of drugs at baseline.

**Conclusions:**

The tissue characteristics of the carotid arterial wall did not change in either the tofogliflozin or conventional treatment group during the 104-week treatment period, and there was no significant difference between the treatment groups.

*Clinical trial registration* UMIN000017607 (https://www.umin.ac.jp/icdr/index.html)

**Supplementary Information:**

The online version contains supplementary material available at 10.1186/s12933-022-01451-6.

## Background

Since sodium-glucose cotransporter 2 (SGLT2) inhibitors have a pleiotropic antiatherogenic effect, they are expected to attenuate the progression of atherosclerosis. Indeed, several studies have shown that SGLT2 inhibitors attenuate arteriosclerosis in animal models of the disease [1–3]. Furthermore, clinical trials in patients with type 2 diabetes mellitus (T2DM) have shown that SGLT2 inhibitors significantly reduce cardiovascular outcomes [4, 5].

Recently, the Using Tofogliflozin for Possible better Intervention against Atherosclerosis for Type 2 Diabetes Patients (UTOPIA) study, a randomized co Tofogliflozin

ntrolled trial was conducted to investigate the preventive effects of tofogliflozin, an SGLT2 inhibitor, on the progression of carotid intima-media thickness (IMT) in patients with apparent CVD-free T2DM, and found that there were no significant differences in the progression of IMT between the tofogliflozin treatment and conventional treatment [6]. However, whether SGLT2 inhibitors affect the tissue characteristics of the human arterial wall remains unclear.

Presently, various modalities such as ultrasonography, computed tomography (CT), magnetic resonance imaging (MRI), and positron emission tomography (PET) are being used to detect vulnerable plaque in coronary and carotid arteries [7–9]. Unfortunately, measurements for carotid atherosclerosis obtained by methods other than ultrasonography were not available for most participants in the UTOPIA study. Recent studies have revealed that noninvasive ultrasonic tissue characterization of carotid plaques using gray-scale median (GSM) reflects plaque composition, and that low-GSM plaques, which consist mainly of lipids, inflammatory infiltrations, and/or hemorrhages, are considered unstable [10]. The aim of the present sub-analysis was to evaluate the effect of tofogliflozin on the longitudinal change in the GSM value, an index of the ultrasonic tissue characteristics of the carotid wall, in patients with T2DM, using data obtained from the UTOPIA trial.

## Methods

### Study design

The present study was a post hoc analysis based on data obtained from the UTOPIA trial, which was a multicenter prospective, randomized, open-label, blinded-endpoint study conducted to evaluate the efficacy of tofogliflozin in preventing the progression of atherosclerosis in patients with T2DM. The study design, study schedule, and outcomes of the original UTOPIA trial have been described in detail previously [11].

In brief, participants eligible for the study were those who had T2DM in whom the target of blood glucose control specified in the Treatment Guide for Diabetes (edited by the Japan Diabetes Society in 2014–2015) was not achieved (glycated hemoglobin [HbA1c] ≥ 6% but < 9%), despite dietary/exercise therapy or concomitant therapeutic drugs for T2DM other than SGLT2 inhibitors, and those aged 30–74 years at the time of enrollment. Exclusion criteria were (1) presence of type 1 or secondary diabetes; (2) being in the perioperative period or having a serious infection or injury; (3) having a history of myocardial infarction, angina, stroke, or cerebral infarction; (4) an estimated glomerular filtration rate (eGFR) < 30 mL/min/1.73 m^2^ or end-stage renal failure, (5) a serious liver functional impairment, (6) moderate to severe heart failure (New York Heart Association stage III or higher), (7) urinary tract or genital infection, (8) being pregnant, possibly pregnant, nursing, or planning to conceive a child; (9) with a history of hypersensitivity to the study drug; (10) presence or history of a malignant tumor (exceptions: patients not on medication for malignant tumor and those without recurrence of the disease and without recurrence risks during this study were allowed to participate), (11) prohibition from using tofogliflozin, and (12) other ineligibility determined by an investigator.

Individuals with CVD-free T2DM who met the above eligibility criteria were asked to participate in this study, and all patients who agreed to participate were enrolled. Originally, a total of 340 patients were enrolled at 22 outpatient diabetes clinics across Japan, and randomly allocated into either the tofogliflozin group (20 mg of tofogliflozin once daily, n = 169) or the conventional treatment group (those using drugs other than the SGLT2 inhibitor) (n = 171).

The protocols of the original study (approval number: N18007, date of approval: 2019/8/7) and this sub-analysis (approval number: 19448, date of approval: 2020/3/24) were approved by the Osaka University Clinical Research Review Committee. On the grounds that the current study was a post-hoc analysis using only existing materials, the study was considered exempt from written informed consent of study participants, in accordance with the Ethical Guidelines for Medical and Health Research Involving Human Subjects in Japan. Instead, relevant information regarding the study was open to the public, and opportunities for refusal were ensured. The study was conducted in accordance with the Declaration of Helsinki, the Ethical Guidelines for Medical and Health Research Involving Human Subjects, the Clinical Trials Act, and other current legal regulations in Japan.

This study was registered in the University Hospital Medical Information Network Clinical Trials Registry, which is a non-profit organization in Japan that meets the requirements of the International Committee of Medical Journal Editors (UMIN000017607).

### Ultrasound examination

B-mode ultrasonography of the carotid artery was performed using an ultrasound machine with a high-frequency linear transducer, according to the guidelines of the Japan Society of Ultrasonics [9]. Scanning of the extracranial common carotid artery (CCA) was performed bilaterally in three different longitudinal projections as well as transverse projections. All scans were electronically stored and sent to the central office for reading by a single experienced reader unaware of the patients’ clinical characteristics in a random order. The IMT was measured as the distance between two parallel echogenic lines corresponding to the blood-intima and media-adventitia interface on the posterior wall of the artery using an automated digital edge-detection software (Intimascope; MediaCross, Tokyo, Japan) [12]. The software system averaged 200 points of IMT values in the segment 2 cm proximal to the dilation of the carotid bulb (mean-IMT-CCA). Localized elevated lesions with a maximum thickness of > 1 mm, having a point of inflection on the surface of the intima-media complex, are defined as “carotid plaque” based on the guideline from the Japan Society of Ultrasonics in Medicine [13]. The presence of plaque lesions, the thickness of the plaque lesions (IMT value), and the histological status of plaque lesions (GSM value) were evaluated independently using images taken at three observation time points: baseline, 52 weeks, and 104 weeks.

**Fig. 1:**
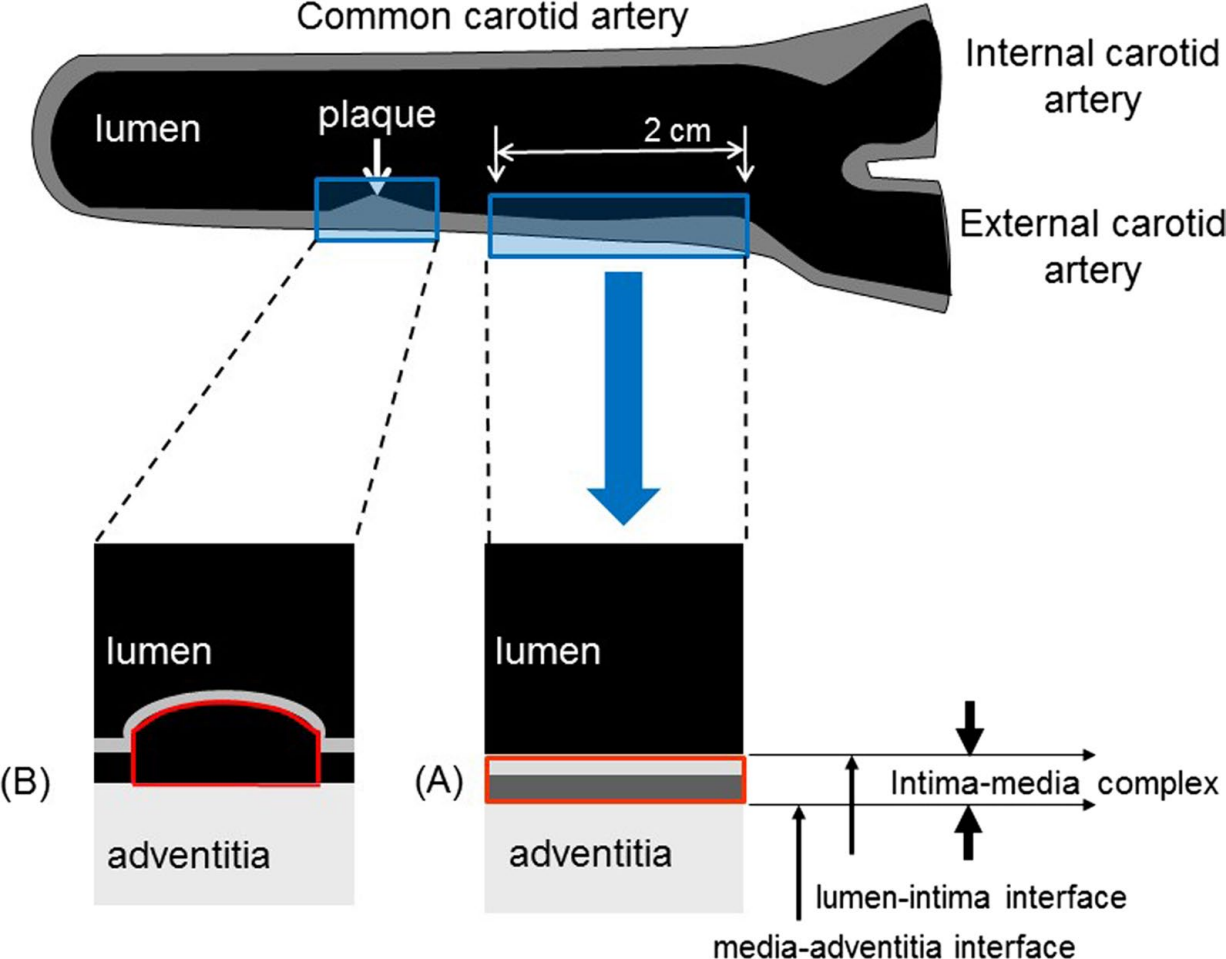
Measurement of GSM values. **A** The right and left mean-IMT-CCA areas (intima-media complex of the segment 2 cm proximal to the dilation of the carotid bulb) were delineated using a freehand tool (shown as a red frame), and the GSM values of the selected area were read from the entire delineated area (“right GSM-CCA” and “left GSM-CCA”). **B** Similarly, if atherosclerotic plaque lesions or thickened (focal IMT ≥ 1.0 mm) lesions were detected, the GSM values of all these lesions were measured using the same method: the lesions were delineated with a freehand tool (shown as a red frame), and the GSM value of each plaque was read from the entire delineated area (“GSM-plaque”)

The echogenicity of the arterial wall was assessed based on the GSM method in a gray-scale range of 0 to 255 (0 as the darkest and 255 as the brightest tone). Adobe Photoshop software (Adobe Systems, version 7.0, San Jose, CA, USA) was used for image standardization and calculation of gray-scale values. In practice, according to a previous report, the standardization of the B-mode image was performed using a curve option so that the GSM for the blood ranged from 0 to 5, and for the adventitia from 185 to 195 [14]. Thus, the gray-scale values of all pixels would change according to the new linear scale defined by the reference values for blood and adventitia, although the ultrasound gain settings for each image were not always standardized. The right and left mean-IMT-CCA areas (intima-media complex of the segment 2 cm proximal to the dilation of the carotid bulb) were then delineated using a freehand tool (shown as a red frame in Fig. 1A), and the GSM values of the selected area were read from the entire delineated area (right GSM-CCA and left GSM-CCA). Subsequently, the average values of the right and left carotid arteries were defined as “mean GSM-CCA.” Similarly, if atherosclerotic plaque lesions or thickened (focal IMT ≥ 1.0 mm) lesions were detected, the GSM values of all these lesions were measured using the same method: the lesions were delineated with a freehand tool (shown as a red frame in Fig. 1B), and the GSM value of each plaque was read from the entire delineated area (GSM-plaque). Subsequently, the lowest value of the right and left carotid arteries were defined as “right GSM-lesion” and “left GSM-lesion,” respectively. Thus, the region size was set for every measurement timing and not standardized across sides and participants.

In this study, repeated GSM measurements were performed in a blinded manner, and the analyses were performed at a core laboratory to avoid bias and measurement errors between institutions, which were electronically stored and sent to the core laboratory for reading by a single experienced reader unaware of the patients’ clinical characteristics in a random order. The same procedure for analyzing carotid GSM values was used in our previous studies [15–17]. Thus, the reliability and reproducibility of the GSM measurements were confirmed in the current study.

### Statistical analysis

All enrolled patients, except those without baseline GSM measurements, were analyzed. As for baseline and follow-up variables, group comparisons were performed using Student’s t-test or the Wilcoxon rank-sum test for continuous variables and Fisher’s exact test or the chi-square test for categorical variables. Primary analysis was performed using the mixed-effects model for repeated measures (MMRM) with treatment group, time (week), interactions between treatment group and time (week), and baseline GSM as fixed effects; an unstructured covariate was used to model the covariance of within-patient variability. The sensitivity analysis assessed differences in delta change in IMT from baseline between the two groups using analysis of covariance (ANCOVA) models that included treatment group, age, sex, baseline GSM, systolic blood pressure, and administration of statins.

All statistical tests were two-sided, with a 5% significance level. All analyses were performed using SAS software (version 9.4; SAS Institute, Cary, NC, USA).

## Results

### Study population

After excluding three patients from further analysis due to missing data for the primary endpoint, 168 and 169 patients in the tofogliflozin and conventional treatment groups were included in the full analysis set, respectively. At baseline, serum triglyceride (TG) levels were slightly but significantly lower in the tofogliflozin treatment group than in the conventional treatment group. The proportion of patients using DPP-4 inhibitors and angiotensin II receptor blockers were significantly lower in the tofogliflozin treatment group than in the conventional treatment group. Regarding the other parameters, there were no significant differences in the baseline characteristics between the two groups (Table 1).

**Table 1:** Clinical characteristics of patients in both treatment groups

Parameters	Tofogliflozin group (n = 168)	Conventional group (n = 169)	P value
Sex (males) (%)	98 (58.3)	98 (58.0)	0.95
Age (years)	61.4 ± 9.3	60.8 ± 9.7	0.60
Current smoking	38 (22.8)	29 (17.2)	0.20
Body mass index (kg/m^2^)	27.0 ± 5.8	27.0 ± 4.6	0.98
Waist circumference (cm)	93.1 ± 12.7	93.7 ± 11.7	0.66
Duration of diabetes (years)	12.1 ± 8.4	12.4 ± 8.2	0.75
HbA1c (%)	7.4 ± 0.7	7.3 ± 0.7	0.22
Fasting blood glucose (mmol/L)	7.8 ± 1.7	7.9 ± 1.8	0.82
Hypertension	87 (51.8)	104 (61.5)	0.07
Systolic blood pressure (mmHg)	133.0 ± 14.5	134.5 ± 17.4	0.39
Diastolic blood pressure (mmHg)	77.7 ± 10.0	79.1 ± 11.0	0.23
Dyslipidemia	106 (63.1)	121 (71.6)	0.10
Total cholesterol (mmol/L)	4.95 ± 0.74	4.93 ± 0.82	0.80
LDL cholesterol (mmol/L)	2.88 ± 0.69	2.89 ± 0.66	0.87
HDL cholesterol (mmol/L)	1.42 ± 0.36	1.37 ± 0.31	0.21
Triglyceride (mmol/L)	1.20 [0.93, 1.78]	1.45 [1.00, 1.89]	0.049
Diabetic retinopathy	28 (16.9)	33 (19.0)	0.60
Diabetic nephropathy	48 (28.6)	52 (30.8)	0.72
eGFR (mL/min/1.73 m^2^)	80.8 ± 20.9	81.9 ± 24.1	0.66
Urinary albumin excretion (mg/g/cre)	13.0 [6.3, 37.0]	17.4 [5.8, 67.9]	0.54
Use of glucose-lowering agents	152 (90.5)	151 (89.3)	0.86
Metformin	91 (54.2)	99 (58.6)	0.44
Sulfonylurea	38 (22.6)	43 (25.4)	0.61
Glinides	10 (6.0)	10 (5.9)	1.00
Thiazolidinediones	18 (10.7)	23 (13.6)	0.51
α-Glucosidase inhibitor	24 (14.3)	25 (14.8)	1.00
DPP-4 inhibitors	74 (44.4)	94 (55.6)	0.039
GLP-1 receptor agonists	23 (13.7)	12 (7.1)	0.05
Insulins	35 (20.8)	36 (21.3)	1.00
Use of antihypertensive drugs	79 (47.0)	95 (56.2)	0.10
Angiotensin-converting enzyme inhibitors	3 (1.8)	5 (3.0)	0.72
Angiotensin II receptor blockers	63 (37.5)	83 (49.1)	0.037
Direct renin inhibitor	2 (1.2)	0 (0.0)	0.25
Calcium channel blocker	47 (28.0)	54 (32.0)	0.48
Diuretic drugs	8 (4.8)	14 (8.3)	0.27
α-Adrenergic receptor antagonist	2 (1.2)	0 (0.0)	0.25
β-Adrenergic receptor antagonist	3 (1.8)	3 (1.8)	1.00
Others	5 (3.0)	10 (5.9)	0.29
Use of lipid-lowering agents	82 (48.8)	99 (58.6)	0.08
Statins	73 (43.5)	83 (49.1)	0.33
Ezetimibe	10 (6.0)	11 (6.5)	1.00
Resins	0 (0.0)	1 (0.6)	1.00
Fibrates	8 (4.8)	6 (3.6)	0.60
Use of antithrombotic agents	17 (10.1)	14 (8.3)	0.58
Antiplatelet agents	15 (8.9)	10 (5.9)	0.31
Anticoagulants	2 (1.2)	4 (2.4)	0.68
Others	0 (0.0)	0 (0.0)	–

Data are presented as number (%) of patients or mean ± SD values or median [25th and 75th percentiles] values

*HbA1c* glycated hemoglobin, *SD* standard deviation, *LDL* low-density lipoprotein, *HDL* high-density lipoprotein, *DPP-4* dipeptidyl peptidase-4, *GLP-1* glucagon-like peptide-1

Among the study participants, 140 and 146 in the tofogliflozin and conventional treatment groups, respectively, completed the allocated treatment regimen. Reductions in HbA1c, BMI, waist circumference, systolic blood pressure, and urinary albumin excretion during the 104-week treatment period were significantly larger in the tofogliflozin group (n = 168) than in the conventional group (n = 169). There were no significant differences in the changes in serum total, high-density lipoprotein cholesterol (HDLC), and low-density lipoprotein cholesterol, TG levels, and eGFR from baseline to 104 weeks between the two groups (Additional file 1: Table S1).

Over the course of the study, DPP-4 inhibitor use was significantly higher, and after 52 weeks, metformin use was significantly higher in the conventional group than in the tofogliflozin group (Additional file 1: Table S2). Furthermore, antihypertensive drugs, especially angiotensin II receptor blockers (ARBs), were significantly more frequently used, and the use of lipid-lowering agents tended to be higher in the conventional group than in the tofogliflozin group during the study (Additional file 1: Table S3).

### Effect of tofogliflozin on the carotid wall

At baseline, mean GSM-CCA and left GSM-CCA values were measurable in all the study participants; however, left GSM-CCA values were not measurable in two participants in the tofogliflozin treatment group. Atherosclerotic plaques and/or thickened (focal IMT ≥ 1.0 mm) lesions were observed in the right CCA in 208 patients (99 and 109 in the tofogliflozin and conventional treatment group, respectively), in the left CCA in 218 patients (107 and 111 in the tofogliflozin and conventional treatment groups, respectively), and in bilateral CCA in 147 patients (65 and 82 in the tofogliflozin and conventional treatment groups, respectively). The GSM values of these plaques were measured, and there were no significant differences in any of the GSM measures (i.e., mean GSM-CCA, right GSM-CCA, left GSM-CCA, right GSM-lesion, and left GSM-lesion) between the two treatment groups at baseline (Table 2).

**Table 2:** Effects of tofogliflozin on gray-scale median values

	Tofogliflozin group	Conventional group	Treatment effect (tofogliflozin-conventional treatment) mean change (95% CI), P value	P value between groups
Mean GSM-CCA				
Baseline	38.07 ± 12.49 (n = 168)	38.42 ± 14.19 (n = 169)		0.81
Week 52	38.09 ± 12.01	39.49 ± 14.29		0.35
Week 104	37.32 ± 12.86	38.63 ± 14.80		0.42
Change at Week 52	− 0.38 ± 12.02	1.03 ± 11.58	− 1.40 (− 4.03, 1.23), P = 0.30	
Change at Week 104	− 0.98 ± 11.15	0.26 ± 11.90	− 1.24 (− 3.87, 1.38), P = 0.35	
Right GSM-CCA				
Baseline	37.91 ± 14.36 (n = 168)	37.90 ± 15.82 (n = 169)		0.99
Week 52	38.01 ± 14.21	39.37 ± 17.12		0.45
Week 104	36.06 ± 13.84	38.33 ± 15.75		0.19
Change at Week 52	− 0.07 ± 16.25	1.45 ± 17.47	− 1.52 (− 5.29, 2.26), P = 0.43	
Change at Week 104	− 1.80 ± 14.35	0.52 ± 15.22	− 2.33 (− 5.70, 1.05), P = 0.18	
Left GSM-CCA				
Baseline	38.06 ± 13.93 (n = 166)	38.94 ± 15.44 (n = 169)		0.58
Week 52	38.26 ± 13.27	39.50 ± 16.22		0.46
Week 104	38.48 ± 15.24	38.84 ± 16.53		0.85
Change at Week 52	− 0.40 ± 13.66	0.44 ± 13.6	− 0.85(− 3.84,2.14), P = 0.58	
Change at Week 104	− 0.06 ± 14.50	0.23 ± 13.81	− 0.29 (− 3.53, 2.95), P = 0.86	
Right GSM-lesion				
Baseline	47.53 ± 25.71 (n = 99)	48.43 ± 22.65 (n = 109)		0.79
Week 52	48.55 ± 26.02	48.93 ± 24.42		0.92
Week 104	46.70 ± 24.00	49.92 ± 24.12		0.36
Change at Week 52	1.33 ± 24.69	− 0.83 ± 25.66	2.15 (− 5.20, 9.50), P = 0.56	
Change at Week 104	0.37 ± 27.09	2.01 ± 28.34	− 1.64 (− 10.01, 6.73), P = 0.70	
Left GSM-lesion				
Baseline	47.93 ± 23.29 (n = 107)	48.24 ± 23.96 (n = 111)		0.92
Week 52	50.76 ± 27.66	46.30 ± 24.60		0.23
Week 104	47.83 ± 22.64	47.65 ± 21.48		0.95
Change at Week 52	4.31 ± 25.42	− 2.68 ± 25.85	6.99 (− 0.34, 14.32), P = 0.06	
Change at Week 104	1.33 ± 21.36	− 0.14 ± 22.96	1.47 (− 4.98, 7.92), P = 0.65	
Common mean-IMT-CCA				
Baseline	0.87 ± 0.16 (n = 168)	0.86 ± 0.15 (n = 169)		0.93
Week 52	0.79 ± 0.14	0.78 ± 0.13		0.76
Week 104	0.74 ± 0.14	0.72 ± 0.13		0.48
Change at Week 52	− 0.085 ± 0.071^§^	− 0.085 ± 0.067^§^	0.001 (− 0.012, 0.015), P = 0.84	
Change at Week 104	− 0.136 ± 0.091^§^	− 0.142 ± 0.080^§^	0.008 (− 0·009, 0·025), P = 0.35	
Right mean-IMT-CCA				
Baseline	0.84 ± 0.15 (n = 168)	0.85 ± 0.15 (n = 169)		0.84
Week 52	0.77 ± 0.13	0.77 ± 0.14		0.96
Week 104	0.72 ± 0.13	0.72 ± 0.14		0.76
Change at Week 52	− 0.074 ± 0.076^§^	− 0.077 ± 0.074^§^	0.003 (0.013, 0.018), P = 0.74	
Change at Week 104	− 0.124 ± 0.092^§^	− 0.131 ± 0.086^§^	0.007 (0.011, 0.025), P = 0.46	
Left mean-IMT-CCA				
Baseline	0.89 ± 0.20 (n = 168)	0.88 ± 0.19 (n = 169)		0.76
Week 52	0.80 ± 0.18	0.79 ± 0.17		0.66
Week 104	0.75 ± 0.19	0.73 ± 0.17		0.38
Change at week 52	− 0.096 ± 0.107^§^	− 0.094 ± 0.092^§^	0.001 (0.019, 0.020), P = 0.95	
Change at week 104	− 0.148 ± 0.127^§^	− 0.153 ± 0.107^§^	0.010 (0.013, 0.033), P = 0.41	

Data are presented as the mean ± SD unless stated otherwise. Comparisons of GSM values during treatment with those at baseline were performed using a one-sample t-test based on the mixed-effects model for repeated measures. *P < 0.05, ^#^P < 0.01, ^§^P < 0.001. Differences in GSM (or IMT) between groups at each point and delta change in GSM (or IMT) from baseline to week 52 and 104 between groups at each point (treatment effect) were analyzed using Student’s t-test

*CCA* common carotid artery, *GSM* Gray-Scale Median, *IMT* intima-media thickness

Both the tofogliflozin treatment and conventional treatment groups did not show significant effects on the mean GSM-CCA values (from 38.07 ± 12.49 to 37.32 ± 12.86 and 38.42 ± 14.19 to 38.63 ± 14.80, respectively), right GSM-CCA values (from 37.91 ± 14.36 to 36.06 ± 13.84 and 37.90 ± 15.82 to 38.33 ± 15.75, respectively), and left GSM-CCA values (from 38.06 ± 13.93 to 38.48 ± 15.24 and 38.94 ± 15.44 to 38.84 ± 16.53, respectively) during the 104-week observation period. There was also no significant difference in right- and left-GSM-lesion values between both groups (Table 2).

The magnitude of the change in GSM values between the two treatment groups during the treatment period was compared using the MMRM (Table 2). These analyses also revealed that neither tofogliflozin treatment nor conventional treatment significantly changed any of the GSM measures. In addition, there was no significant difference in the change in GSM measures from baseline to week 52 and week 104, between the two groups. Similar findings were observed even after adjustment for possible confounding factors such as age, sex, BMI, HbA1c, serum lipid levels (e.g., total cholesterol, HDLC, and TG), blood pressure, smoking status, and administration of anti-diabetic, anti-hypertensive, anti-hyperlipidemic, and anti-platelet drugs (Table 3).

**Table 3:** Effects of tofogliflozin on the GSM values of common carotid arteries after adjusting for confounders

	Model 1	Model 2	Model 3	Model 4	Model 5	Model 6	Model 7
Mean GSM-CCA							
Week 52	− 1.43 (− 3.78, 0.91)	− 1.40 (− 3.75, 0.94)	− 1.80 (− 4.23, 0.63)	− 2.00 (− 4.46, 0.47)	− 2.13 (− 4.60, 0.33)	− 1.86 (− 4.33, 0.60)	− 1.88 (− 4.38, 0.62)
Week 104	− 1.19 (− 3.59, 1.22)	− 1.16 (− 3.57, 1.26)	− 1.03 (− 3.49, 1.43)	− 1.21 (− 3.68, 1.25)	− 1.36 (− 3.83, 1.10)	− 1.08 (− 3.56, 1.41)	− 1.12 (− 3.64, 1.41)
Right GSM-CCA							
Week 52	− 1.55 (− 4.79, 1.68)	− 1.48 (− 4.71, 1.75)	− 1.76 (− 5.14, 1.61)	− 2.15 (− 5.58, 1.27)	− 2.30 (− 5.74, 1.13)	− 1.98 (− 5.42, 1.46)	− 2.30 (− 5.75, 1.14)
Week 104	− 2.13 (− 5.02, 0.76)	− 2.06 (− 4.95, 0.83)	− 1.89 (− 4.88, 1.09)	− 2.25 (− 5.24, 0.75)	− 2.40 (− 5.39, 0.59)	− 2.07 (− 5.08, 0.94)	− 2.45 (− 5.49, 0.60)
Left GSM-CCA							
Week 52	− 1.07 (− 3.75, 1.61)	− 1.10 (− 3.79, 1.58)	− 1.54 (− 4.35, 1.26)	− 1.69 (− 4.55, 1.17)	− 1.81 (− 4.66, 1.04)	− 1.54 (− 4.39, 1.31)	− 1.29 (− 4.19, 1.61)
Week 104	− 0.40 (− 3.38, 2.59)	− 0.43 (− 3.42, 2.57)	− 0.26 (− 3.33, 2.82)	− 0.39 (− 3.48, 2.70)	− 0.54 (− 3.63, 2.56)	− 0.26 (− 3.36, 2.85)	− 0.02 (− 3.15, 3.11)
Right GSM-lesion							
Week 52	1.32 (− 4.81, 7.45)	1.34 (− 4.80, 7.48)	0.01 (− 6.31, 6.34)	− 0.74 (− 7.18, 5.70)	0.61 (− 7.07, 5.84)	0.01 (− 6.46, 6.47)	− 0.49 (− 6.98, 5.99)
Week 104	− 2.68 (− 9.58, 4.22)	− 2.63 (− 9.52, 4.26)	− 3.30 (− 10.51, 3.91)	− 4.02 (− 11.33, 3.30)	− 3.90 (− 11.28, 3.47)	− 3.27 (− 10.48, 3.94)	− 3.81 (− 10.94, 3.32)
Left GSM-lesion							
Week 52	5.77 (− 0.67, 12.22)	5.83 (− 0.50, 12.16)	5.83 (− 0.73, 12.38)	6.18 (− 0.57, 12.93)	5.76 (− 1.07, 12.59)	5.83 (− 0.87, 12.53)	6.58 (− 0.27, 13.44)
Week 104	0.83 (− 4.73, 6.39)	1.03 (− 4.53, 6.60)	0.67 (− 5.00, 6.34)	0.99 (− 4.86, 6.85)	0.54 (− 5.40, 6.49)	0.61 (− 5.20, 6.43)	1.45 (− 4.40, 7.30)

Treatment effect (tofogliflozin and conventional treatment) is expressed as the mean change (95% CI). Differences in delta change in GSM from baseline at 52 and 104 weeks between groups at each point (treatment effect) were analyzed using a mixed-effects model for repeated measures. *P < 0.05, ^#^P < 0.01, ^§^P < 0.001

Model 1: treatment group, week, interactions between treatment groups and week, and baseline GSM were included as fixed effects. Model 2: model 1 plus sex and age were included as fixed effects

Model 3: model 2 plus body mass index, HbA1c, total cholesterol, high-density lipoprotein-cholesterol, triglyceride, and systolic blood pressure at baseline were included as fixed effects

Model 4: model 3 plus smoking, DPP-4 inhibitors, and angiotensin II receptor blockers at baseline were included as fixed effects. Model 5: model 4 plus statin and anti-platelets at baseline were included as fixed effects

Model 6: model 3 plus smoking, hypoglycemic agents, antihypertensive agents, antihyperlipidemic agents, and antiplatelets at baseline were included as fixed effects

Model 7: model 3 plus smoking, metformin, sulfonylureas, glinides, pioglitazone, α-glucosidase inhibitors, GLP-1 receptor agonists, DPP-4 inhibitors, antihypertensive agents, antihyperlipidemic agents, and antiplatelets at baseline were included as fixed effects

*CCA* common carotid artery, *GSM* Gray-Scale Median

Regarding the effect of tofogliflozin on mean-IMT-CCA, the results were similar to those observed in the original UTOPIA trial: we confirmed statistically significant IMT reduction in both the tofogliflozin treatment and the control groups, and that there were no significant differences in the progression in mean-IMT-CCA between the two treatment groups (Table 2).

In addition, we have added a sub-analysis, where the changes of GSM-CCA were compared among the three groups based on tertiles of changes in mean-IMT during the treatment period. This analysis also showed no significant differences in the mean GSM-CCA from baseline to week 104 among them (Additional file 1: Table S4). Similar results were observed in the analyses where the study participants were divided into the tofogliflozin treatment group and the conventional treatment group (Additional file 1: Table S4).

## Discussion

Several previous studies have shown that SGLT2 inhibitors, such as empagliflozin and canagliflozin, attenuated arteriosclerosis in mouse models of atherosclerotic disease [1–3]. In addition, it has been reported that carotid IMT reduced after treatment with SGLT2 inhibitors in patients with T2DM [6, 18]. However, whether SGLT2 inhibitors affect the tissue characteristics of the arterial wall remains unclear. Therefore, using data obtained from the UTOPIA trial, a randomized controlled trial conducted to investigate the preventive effects of tofogliflozin on the progression of carotid IMT in patients with apparent CVD-free T2DM, we evaluated the longitudinal change in the ultrasonic tissue characteristics of the carotid wall and found that tofogliflozin treatment did not significantly affect the tissue characteristics of the carotid wall.

Notably, the original study clearly demonstrated that carotid IMT significantly reduced after 104 weeks of tofogliflozin treatment, while there were no significant differences in the reduction in IMT between the two treatment groups [6].

One possible explanation for this discrepancy between the effect of tofogliflozin on carotid IMT and that on GSM is that the determinants of arterial thickening in the carotid and those of the tissue characteristics of the carotid wall are not the same. Many studies have shown that HbA1c and blood pressure, as well as male sex, age, BMI, and serum HDLC levels are major risk factors for carotid IMT progression [19–22]. On the other hand, our previous study revealed that low HDLC levels and high BMI were major determinants of low GSM values in patients with diabetes and that HbA1c and blood pressure levels were not associated with carotid GSM [23]. Similarly, Andersson et al. reported that HDLC and BMI were independent determinants of carotid GSM, while blood pressure, smoking, and BMI were independent determinants of carotid IMT [24]. Generally, lipid oxidation and inflammation, rather than hyperglycemia and hypertension, are considered the most critical determinants of arterial echogenicity [25–27]. In our study, reductions in HbA1c and systolic blood pressure levels as well as a reduction in BMI and an elevation of HDLC levels were observed in the tofogliflozin treatment group (Additional file 1: Table S1). It is possible that such a broad and potent effect of tofogliflozin on the major determinants of carotid IMT, including hyperglycemia and hypertension, as well as dyslipidemia, induced a substantial reduction of IMT. However, the relatively small effect of tofogliflozin on the lipid-related parameters was not enough to induce a change in the tissue characteristics.

It is also possible that the beneficial effects of tofogliflozin on the tissue characteristics of the carotid wall, if any, was masked by the administration of additional antidiabetic, antilipidemic, and antihypertensive agents, which are supposed to affect the tissue characteristics of the arterial wall. In particular, lipid-lowering agents have a potent anti-atherogenic effect and have been reported to improve the tissue characteristics of plaques in the carotid artery [28–30]. Notably, the administration rate of lipid-lowering agents during the treatment period was relatively higher in the conventional treatment group than in the tofogliflozin treatment group; the proportions of lipid-lowering agent users were 48.8% and 58.6% in the tofogliflozin and conventional treatment groups at baseline (p = 0.08), respectively, and 52.9% and 63.2% at 104 weeks (p = 0.08), respectively (Additional file 1: Table S3). Such an uncontrolled imbalance in the administration of lipid-lowering agents might have masked the potential beneficial effects of tofogliflozin. Similarly, it is possible that the administration of DPP-4 inhibitors, which was more frequent in the control group during the treatment period (Additional file 1: Table S2), may have affected the outcomes, since DPP-4 inhibitors also affect the tissue characteristics of the carotid artery [15, 16].

Peppa-Patrikiou et al. reported an increase in carotid IMT in participants with insulin-dependent diabetes mellitus and that IMT was positively related to urinary free cortisol in this population. Their findings indicated that hyperactivity of the adrenals might contribute to macroangiopathy via mechanisms such as stress, hypoglycemic episodes, and increased endothelin production [31]. Other researchers have also reported that the progression of carotid atherosclerosis was associated with serum or urinary cortisol levels [32–34]. Interestingly, a recent study revealed that administration of tofogliflozin decreased both serum ACTH and cortisol levels in patients with T2DM, indicating that tofogliflozin influences the hypothalamic–pituitary–adrenal pathway in this population [35]. Therefore, SGLT2 inhibitors may be related to the attenuation of carotid atherosclerosis via reduction of serum cortisol levels. However, it is unclear whether the effects of tofogliflozin on carotid IMT and GSM could be explained by the serum cortisol level since it was not measured in the UTOPIA trial.

Our study has some limitations. First, this was a post-hoc analysis using data obtained from the UTOPIA trial. It might have lacked the power to detect a smaller effect, which might be the reason why this study did not show a significant difference between the two treatment groups. Prespecified studies with large sample size would be necessary to confirm our findings. Second, the diagnostic performance of ultrasonography is limited due to a user-dependent methodology; however, ultrasonography represents the first-line imaging modality in the evaluation of carotid artery plaques since it is relatively simple, inexpensive, and widely available. Recent advances in medical imaging have enabled noninvasive identification of the characteristics of the carotid wall using other modalities. For example, high-resolution MRI has been used to evaluate the characteristics of carotid atherosclerotic plaques with high accuracy [36], despite being complex, expensive, and less readily available than other imaging modalities. Multi detector CT scanners (MDCT), which enable fast and accurate acquisition of vascular structures with minimal discomfort to the patient, can provide comparable results in the detection of soft tissue subcomponents of the plaque when compared to MRI [37–39]; however, MDCT has also the limitations such as renal toxicity related to the use of contrast medium, radiation exposure, and artifacts. F-18-fluorodeoxyglucose (FDG)-PET can illuminate metabolically active processes and distinguish vulnerable from non-vulnerable plaques, although it does require longer procedural times than other imaging options. In the UTOPIA study, the measurements for carotid atherosclerosis obtained by modalities such as MRI, MDCT, or FDG-PET were not available for most participants. Third, the ultrasound settings for each image were not always standardized. However, the blood was used as the reference for black and the adventitia as the reference for white, and gain settings for measurements within an individual were similar throughout the study. Therefore, the impact of the gain of the ultrasound beam on the GSM value would be quite small, if any. Fourth, regarding the assessment of mean-GSM-CCA, the same regions were measured throughout the study in each patient. However, regarding the assessment of plaque lesions, this study evaluatesthe effect of tofogliflozin administration on the histological status of plaque lesions on a patient-by-patient basis using those lesions with the highest risk at each observation point as the representative value for the given patient. Therefore, the plaque lesions evaluated at the three observation time points (baseline, 52 weeks, and 104 weeks) are not necessarily always the same, and due to the inability to track changes in individual plaque lesions over time, it is possible that the effect of tofogliflozin administration on the histological status of plaque lesions could not be appropriately evaluated. Fifth, the administrations of anti-diabetic, anti-hyperlipidemic, and anti-hypertensive drugs, which may affect the plaque components, were not matched completely. It is possible that the inhibition of atherosclerotic change following tofogliflozin treatment might have been masked by the analogous effects of other drugs used in managing diabetes, as described above. Finally, the participants in this study were Japanese patients with T2DM, a cohort with relatively low cardiovascular risk. Therefore, it would be premature to generalize our findings to other racial or ethnic groups.

## Conclusions

In conclusion, this post hoc sub-analysis suggests that the tissue characteristics of the carotid arterial wall did not change in either the tofogliflozin treatment group or conventional treatment group during the 104-week treatment period and that there was no significant difference between the treatment groups.

## Supplementary Information

**Additional file 1:** **Tables S1.** Between-group comparison of changes in clinical parameters during the treatment period. **Table S2.** Changes in concomitantly used anti-diabetic agents. **Table S3.** Changes in concomitantly used cardiovascular medications. **Table S4.** The changes of GSM-CCA on the basis of tertiles of changes in mean-IMT during observation period

## Data Availability

The datasets generated and/or analyzed during our study will be available from the corresponding author upon reasonable request.

## Abbreviations

ANCOVA: Analysis of covariance
BMI: Body mass index
CVD: Cardiovascular disease
CVOT: Cardiovascular outcome trial
DPP-4: Dipeptidyl peptidase-4
eGFR: Estimated glomerular filtration rate
HbA1c: Glycated hemoglobin
HDLC: High-density lipoprotein cholesterol
IMT: Intima-media thickness
SGLT2: Sodium-glucose cotransporter 2
UTOPIA: Using tofogliflozin for possible better intervention against atherosclerosis for type 2 diabetes patients
