# Supplementary material for: Evaluation of the effect of tofogliflozin on the tissue characteristics of the carotid wall—a sub-analysis of the UTOPIA trial
Source: Cardiovasc Diabetol. 2022 Feb 5;21:19. doi: 10.1186/s12933-022-01451-6 (PMC8817596; doi:10.1186/s12933-022-01451-6)
Supplement: Supplementary file 1 — Additional file 1: Tables S1. Between-group comparison of changes in clinical parameters during the treatment period. Table S2. Changes in concomitantly used anti-diabetic agents. Table S3. Changes in concomitantly used cardiovascular medications. Table S4. The changes of GSM-CCA on the basis of tertiles of changes in mean-IMT during observation period [file 12933_2022_1451_MOESM1_ESM.docx]

**ADDITIONAL FILE 1: MATERIALS**

**UTOPIA trial site investigators (listed in alphabetical order):**

*Hayashi Clinic:* I Hayashi

*Ikeda Municipal Hospital:* M Tsugawa

*Jiyugaoka Medical Clinic:* H Yokoyama

*Juntendo Tokyo Koto Geriatric Medical Center:* H Yoshii

*Juntendo University Graduate School of Medicine:* K Komiyama, T Mita, T Shimizu

*Kansai Rosai Hospital:* T Yamamoto

*Kanda Naika Clinic:* S Kawashima

*Kawasaki Hospital:* T Nakamura

*Kawasaki Medical School:* S Kamei, T Kinoshita, M Shimoda

*Kitasenri Maeda Clinic:* K Maeda

*Kosugi Medical Clinic:* K Kosugi

*Misaki Naika Clinic:* H Yoshii

*NakaKinen Clinic:* H Ishida, T Osonoi, M Saito, A Tamazawa

*Nissay Hospita:* S Sumitani

*Osaka General Medical Center:* N Fujiki, Y Fujita, S Shimizu, Y Umayahara

*National Hospital Organization Osaka National Hospital:* K Kato

*Osaka Police Hospital:* Y Irie, R Kataoka, T Yasuda

*Osaka Rosai Hospital:* Y Kiyohara, M Ohashi, K Ryomoto, Y Takahi

*Osaka University Graduate School of Medicine:* Y Fujishima, Y Fujita, A Fukuhara, K Fukui, Y Hosokawa, A Imagawa, H Iwahashi, K Mukai, N Katakami, T Katsura, D Kawamori, T Kimura, S Kobayashi, J Kozawa, F Kubo, N Maeda, T Matsuoka,K Miyashita, S Nakata, H Ninomiya, H Nishizawa, Y Okuno, M Otsuki, F Sakamoto, S Sasaki, I Sato, N Shimo, I Shimomura, M Takahara, T Takano, A Tokunaga, S Uno, M Yamaoka, S Yoneda

*Otoshi Medical Clinic:* K Ohtoshi

*Shiraiwa Medical Clinic:* T Shiraiwa

*University of Occupational and Environmental Health, Japan:* M Hajime, K Koikawa, F Kuno, A Kurozumi, K Matsushita, M Narisawa, K Tanaka, K Sugai, Y Okada, K Torimoto

**Additional file 1: Table S1. B****etween-group comparison of changes in clinical parameters during the treatment period**

| Parameters | Tofogliflozin group | Conventional group | p value |
| --- | --- | --- | --- |
| Body mass index at baseline (kg/m^2^) | 27.0 ± 5.8 (n = 167) | 27.0 ± 4.6 (n = 168) | 0.98 |
| Week 26 (change from baseline) | –0.7 ± 1.0 (n = 162)^§^ | 0.0 ± 1.2 (n = 163) | < 0.001 |
| Week 52 (change from baseline) | –0.8± 1.3 (n = 159)^§^ | –0.1 ± 1.4 (n = 158) | < 0.001 |
| Week 78 (change from baseline) | –0.8 ± 1.5 (n = 153)^§^ | 0.0 ± 1.5 (n = 156) | < 0.001 |
| Week 104 (change from baseline) | –1.0 ± 1.4 (n = 153)^§^ | –0.2 ± 1.8 (n = 153) | < 0.001 |
| Waist circumference at baseline (cm) | 93.1 ± 12.7 (n = 148) | 93.7 ± 11.7 (n = 153) | 0.66 |
| Week 26 (change from baseline) | –2.0 ± 6.0 (n = 127)^§^ | 1.0 ± 4.3 (n = 126)^#^ | <0.001 |
| Week 52 (change from baseline) | –0.9 ± 6.1 (n = 127) | 1.4 ± 4.8 (n = 134)^§^ | <0.001 |
| Week 78 (change from baseline) | –1.3 ± 6.6 (n = 116)^*^ | 1.4 ± 4.3 (n = 123)^§^ | <0.001 |
| Week 104 (change from baseline) | –1.2 ± 6.0 (n = 123)^*^ | 1.5 ± 4.3 (n = 124)^§^ | <0.001 |
| HbA1c at baseline (%) | 7.4 ± 0.7 (n = 168) | 7.3 ± 0.7 (n = 169) | 0.22 |
| HbA1c at baseline (mmol/mol) | 57.5 ± 8.0 (n = 168) | 56.4 ± 7.8 (n = 169) | 0.22 |
| Week 26 (change from baseline) | –4.4 ± 6.3 (n = 164)^§^ | 0.5 ± 5.7 (n = 164) | < 0.001 |
| Week 52 (change from baseline) | –3.6 ± 7.5 (n = 160)^§^ | –0.4 ± 6.2 (n = 161) | <0.001 |
| Week 78 (change from baseline) | –3.6 ± 8.3 (n = 153)^§^ | 0.0 ± 8.0 (n = 158) | <0.001 |
| Week 104 (change from baseline) | –3.5 ± 8.5 (n = 155)^§^ | 0.6 ± 7.5 (n = 152) | <0.001 |
| Fasting blood glucose at baseline (mmol/L) | 7.8 ± 1.7 (n = 166) | 7.9 ± 1.8 (n = 167) | 0.82 |
| Week 26 (change from baseline) | –0.9 ± 1.6 (n = 154)^§^ | 0.2 ± 2.0 (n = 151) | < 0.001 |
| Week 52 (change from baseline) | –0.8 ± 1.7 (n = 149)^§^ | –0.1 ± 1.8 (n = 153) | 0.001 |
| Week 78 (change from baseline) | –0.5 ± 1.7 (n = 144)^#^ | 0.1 ± 1.9 (n = 145) | 0.008 |
| Week 104 (change from baseline) | –0.7 ± 1.9 (n = 147)^§^ | 0.1 ± 1.8 (n = 148) | <0.001 |
| Total cholesterol at baseline (mmol/L) | 4.95 ± 0.74 (n = 164) | 4.93 ± 0.82 (n = 162) | 0.80 |
| Week 26 (change from baseline) | 0.09 ± 0.55 (n = 155) | –0.02 ± 0.54 (n = 149) | 0.08 |
| Week 52 (change from baseline) | 0.08 ± 0.59 (n = 156) | –0.05 ± 0.57 (n = 155) | 0.06 |
| Week 78 (change from baseline) | 0.09 ± 0.57 (n = 145) | –0.05 ± 0.65 (n = 143) | 0.05 |
| Week 104 (change from baseline) | 0.10 ± 0.69 (n = 150) | –0.05 ± 0.67 (n = 146) | 0.06 |
| LDL cholesterol at baseline (mmol/L) | 2.88 ± 0.69 (n = 167) | 2.89 ± 0.66 (n = 168) | 0.25 |
| Week 26 (change from baseline) | 0.02 ± 0.52 (n = 161) | –0.01 ± 0.51 (n = 161) | 0.53 |
| Week 52 (change from baseline) | 0.01 ± 0.51 (n = 158) | –0.03 ± 0.51 (n = 161) | 0.47 |
| Week 78 (change from baseline) | 0.04 ± 0.50 (n = 152) | –0.05 ± 0.59 (n = 156) | 0.14 |
| Week 104 (change from baseline) | 0.01 ± 0.62 (n = 153) | –0.07 ± 0.57 (n = 152) | 0.28 |
| HDL cholesterol at baseline (mmol/L) | 1.42 ± 0.36 (n = 168) | 1.37 ± 0.31 (n = 169) | 0.21 |
| Week 26 (change from baseline) | 0.07 ± 0.18 (n = 162)^§^ | 0.00 ± 0.18 (n = 161) | 0.001 |
| Week 52 (change from baseline) | 0.06 ± 0.16 (n = 160)^§^ | 0.01 ± 0.18 (n = 161) | 0.008 |
| Week 78 (change from baseline) | 0.08 ± 0.22 (n = 153)^§^ | 0.03 ± 0.20 (n = 158) | 0.017 |
| Week 104 (change from baseline) | 0.08 ± 0.23 (n = 155)^§^ | 0.04 ± 0.20 (n = 153)^*^ | 0.08 |
| Triglyceride at baseline (mmol/L) | 1.20 (0.93, 1.78) (n = 166) | 1.45 (1.00, 1.89) (n = 167) | 0.049 |
| Week 26 (change from baseline) | –0.03 (–0.34, 0.24) (n = 151) | 0.03 (–0.23, 0.36) (n = 149) | 0.22 |
| Week 52 (change from baseline) | –0.03 (–0.24, 0.23) (n = 148) | –0.03 (–0.37, 0.20) (n = 153) | 0.37 |
| Week 78 (change from baseline) | –0.01 (–0.30, 0.23) (n = 144) | –0.01 (–0.34, 0.31) (n = 144) | 0.82 |
| Week 104 (change from baseline) | –0.04 (–0.36, 0.25) (n = 146) | –0.03 (–0.36, 0.29) (n = 147) | 0.92 |
| Systolic blood pressure (mmHg) | 133.0 ± 14.5 (n = 164) | 134.5 ± 17.4 (n = 164) | 0.39 |
| Week 26 (change from baseline) | –4.6 ± 13.9 (n = 159)^§^ | –0.7 ± 15.3 (n = 158) | 0.019 |
| Week 52 (change from baseline) | –6.0 ± 13.0 (n = 156)^§^ | –2.7 ± 17.4 (n = 156) | 0.06 |
| Week 78 (change from baseline) | –5.6 ± 15.4 (n = 150)^§^ | –1.5 ± 18.4 (n = 151) | 0.036 |
| Week 104 (change from baseline) | –5.3 ± 16.4 (n = 149)^§^ | 0.6 ± 18.1 (n = 147) | 0.004 |
| Diastolic blood pressure (mmHg) | 77.7 ± 10.0 (n = 164) | 79.1 ± 11.0 (n = 164) | 0.23 |
| Week 26 (change from baseline) | –1.8 ± 8.9 (n = 159)^#^ | 0.1 ± 9.0 (n = 158) | 0.06 |
| Week 52 (change from baseline) | –3.3 ± 9.4 (n = 156)^§^ | –0.8± 10.1 (n = 156) | 0.027 |
| Week 78 (change from baseline) | –2.8 ± 9.4 (n = 150)^§^ | –1.0 ± 9.8 (n = 151) | 0.10 |
| Week 104 (change from baseline) | –3.3 ± 10.1 (n = 149)^§^ | –0.9 ± 9.9 (n = 147) | 0.037 |
| eGFR (mL/min/1.73 m^2^) | 80.8 ± 20.9 (n = 167) | 81.9 ± 24.1 (n = 168) | 0.66 |
| Week 26 (change from baseline) | –2.9 ± 9.1 (n = 160)^§^ | –2.8 ± 9.4 (n = 161)^#^ | 0.94 |
| Week 52 (change from baseline) | –2.7 ± 9.4 (n = 158)^§^ | –2.3 ± 9.6 (n = 160)^#^ | 0.67 |
| Week 78 (change from baseline) | –3.2 ± 9.9 (n = 153)^§^ | –3.7 ± 10.4 (n = 156) | 0.69 |
| Week 104 (change from baseline) | –3.1 ± 12.2 (n = 154)^#^ | –3.7 ± 10.5 (n = 152)^§^ | 0.63 |
| UAE at baseline (mg/g/cre) | 13.0 (6.3, 37.0) (n = 157) | 17.4 (5.8, 67.9) (n = 156) | 0.54 |
| Week 26 (change from baseline) | –0.2 (–8.9, 6.7) (n = 138) | –0.4 (–9.3, 4.5) (n = 133) | 0.87 |
| Week 52 (change from baseline) | –1.7 (–8.1, 3.9) (n = 145)^*^ | 0.1 (–5.2, 9.6) (n = 145) | 0.033 |
| Week 78 (change from baseline) | –0.8 (–8.3, 8.0) (n = 130) | 1.1 (–5.7, 13.9) (n = 129) | 0.06 |
| Week 104 (change from baseline) | –1.3 (–10.6, 5.6) (n = 143) | 1.8 (–3.8, 20.5) (n = 139)^#^ | 0.006 |

Data are presented as mean ± SD or median (25^th^ and 75^th^ percentiles) values. Differences in parameters between groups at baseline were analyzed using Student’s *t*-test or the Wilcoxon rank-sum test. Differences in parameters from baseline to weeks 26, 52, 78, and 104 within each group were analyzed using a one-sample *t*-test or the Wilcoxon signed-rank test. Differences in parameters from baseline to weeks 26, 52, 78, and 104 between groups were analyzed using Student’s *t*-test or the Wilcoxon rank-sum test. ^*^p < 0.05, ^#^ p < 0.01, ^§^ p < 0.001.

eGFR, estimated glomerular filtration rate; SD: standard deviation; UAE, urinary albumin excretion; HbA1c: glycated hemoglobin; LDL: low-density lipoprotein; HDL: high-density lipoprotein.

**Additional file 1: Table S2. Changes in concomitantly used anti-diabetic agents**

| **Parameters** | **Tofogliflozin treatment group** | **Conventional treatment group** | **P value** |
| --- | --- | --- | --- |
| Any concomitantly used anti-diabetic agents* |  |  |  |
| Baseline | 152 (90.5) | 151 (89.3) | 0.86 |
| Week 26 | 149 (89.8) | 154 (91.7) | 0.58 |
| Week 52 | 141 (88.7) | 148 (91.4) | 0.46 |
| Week 78 | 138 (88.5) | 148 (92.5) | 0.25 |
| Week 104 | 136 (87.7) | 143 (92.3) | 0.26 |
| Metformin |  |  |  |
| Baseline | 91 (54.2) | 99 (58.6) | 0.44 |
| Week 26 | 90 (54.2) | 104 (61.9) | 0.18 |
| Week 52 | 84 (52.8) | 104 (64.2) | 0.042 |
| Week 78 | 81 (51.9) | 103 (64.4) | 0.030 |
| Week 104 | 81 (52.3) | 103 (66.5) | 0.015 |
| Sulfonylurea |  |  |  |
| Baseline | 38 (22.6) | 43 (25.4) | 0.61 |
| Week 26 | 34 (20.5) | 43 (25.6) | 0.30 |
| Week 52 | 35 (22.0) | 46 (28.4) | 0.20 |
| Week 78 | 34 (21.8) | 41 (25.6) | 0.43 |
| Week 104 | 31 (20.0) | 41 (26.5) | 0.23 |
| Glinides |  |  |  |
| Baseline | 10 (6.0) | 10 (5.9) | 1.00 |
| Week 26 | 9 (5.4) | 11 (6.5) | 0.82 |
| Week 52 | 7 (4.4) | 9 (5.6) | 0.80 |
| Week 78 | 8 (5.1) | 10 (6.3) | 0.81 |
| Week 104 | 9 (5.8) | 9 (5.8) | 1.00 |
| Thiazolidinediones |  |  |  |
| Baseline | 18 (10.7) | 23 (13.6) | 0.51 |
| Week 26 | 17 (10.2) | 24 (14.3) | 0.32 |
| Week 52 | 18 (11.3) | 24 (14.8) | 0.41 |
| Week 78 | 17 (10.9) | 22 (13.8) | 0.50 |
| Week 104 | 17 (11.0) | 20 (12.9) | 0.73 |
| α-glucosidase inhibitors |  |  |  |
| Baseline | 24 (14.3) | 25 (14.8) | 1.00 |
| Week 26 | 25 (15.1) | 27 (16.1) | 0.88 |
| Week 52 | 24 (15.1) | 23 (14.2) | 0.88 |
| Week 78 | 25 (16.0) | 24 (15.0) | 0.88 |
| Week 104 | 24 (15.5) | 25 (16.1) | 1.00 |
| DPP-4 inhibitors |  |  |  |
| Baseline | 74 (44.0) | 94 (55.6) | 0.039 |
| 26 weeks | 71 (42.8) | 97 (57.7) | 0.009 |
| 52 weeks | 71 (44.7) | 92 (56.8) | 0.034 |
| 78 weeks | 70 (44.9) | 91 (56.9) | 0.043 |
| 104 weeks | 67 (43.2) | 88 (56.8) | 0.023 |
| GLP-1 R agonists |  |  |  |
| Baseline | 23 (13.7) | 12 (7.1) | 0.05 |
| Week 26 | 23 (13.9) | 14 (8.3) | 0.12 |
| Week 52 | 22 (13.8) | 16 (9.9) | 0.30 |
| Week 78 | 21 (13.5) | 14 (8.8) | 0.21 |
| Week 104 | 21 (13.5) | 13 (8.4) | 0.20 |
| Insulins |  |  |  |
| Baseline | 35 (20.8) | 36 (21.3) | 1.00 |
| Week 26 | 34 (20.5) | 36 (21.4) | 0.89 |
| Week 52 | 32 (20.1) | 36 (23.5) | 0.50 |
| Week 78 | 30 (19.2) | 37 (23.1) | 0.41 |
| Week 104 | 30 (19.4) | 35 (22.6) | 0.58 |

Data are number (%) of patients. The two treatment groups were compared by Fisher’s exact test. *; Administration of tofogliflozin in the tofogliflozin treatment group was not counted as concomitantly used anti-diabetic agents.

**Additional file 1: Table S3. Changes in concomitantly used cardiovascular medications**

|  | **Tofogliflozin treatment group** | **Conventional treatment group** | **P value** |
| --- | --- | --- | --- |
| Anti-hypertensive drugs |  |  |  |
| Any antihypertensive drugs |  |  |  |
| Baseline | 79 (47.0) | 95 (56.2) | 0.10 |
| Week 26 | 78 (47.0) | 97 (57.7) | 0.06 |
| Week 52 | 75 (47.2) | 96 (59.3) | 0.034 |
| Week 78 | 73 (46.8) | 95 (59.4) | 0.032 |
| Week 104 | 73 (47.1) | 91 (58.7) | 0.05 |
| Angiotensin-converting enzyme inhibitors |  |  |  |
| Baseline | 3 (1.8) | 5 (3.0) | 0.72 |
| Week 26 | 3 (1.8) | 5 (3.0) | 0.72 |
| Week 52 | 2 (1.3) | 5 (3.1) | 0.45 |
| Week 78 | 3 (1.9) | 5 (3.1) | 0.72 |
| Week 104 | 3 (1.9) | 5 (3.2) | 0.72 |
| Angiotensin II receptor blockers |  |  |  |
| Baseline | 63 (37.5) | 83 (49.1) | 0.037 |
| Week 26 | 62 (37.3) | 84 (50.0) | 0.021 |
| Week 52 | 61 (38.4) | 82 (50.6) | 0.033 |
| Week 78 | 58 (37.2) | 81 (51.9) | 0.009 |
| Week 104 | 56 (36.1) | 79 (51.0) | 0.012 |
| Calcium channel blockers |  |  |  |
| Baseline | 47 (28.0) | 54 (32.0) | 0.48 |
| Week 26 | 45 (27.1) | 54 (32.1) | 0.34 |
| Week 52 | 45 (28.3) | 56 (34.6) | 0.23 |
| Week 78 | 44 (28.2) | 57 (35.6) | 0.18 |
| Week 104 | 45 (29.0) | 56 (36.1) | 0.23 |
| Lipid-lowering agents |  |  |  |
| Any lipid-lowering agents |  |  |  |
| Baseline | 82 (48.8) | 99 (58.6) | 0.08 |
| Week 26 | 81 (48.8) | 99 (58.9) | 0.08 |
| Week 52 | 76 (47.8) | 99 (61.1) | 0.019 |
| Week 78 | 80 (51.3) | 99 (61.9) | 0.07 |
| Week 104 | 82 (52.9) | 98 (63.2) | 0.08 |
| Statins |  |  |  |
| Baseline | 73 (43.5) | 83 (49.1) | 0.33 |
| Week 26 | 72 (43·4) | 83 (49.4) | 0.28 |
| Week 52 | 67 (42.1) | 84 (51.9) | 0.09 |
| Week 78 | 70 (44.9) | 87 (54.4) | 0.09 |
| Week 104 | 73 (47.1) | 87 (56.1) | 0.14 |
| Antithrombotic agents |  |  |  |
| Any anti-thrombotic agents |  |  |  |
| Baseline | 17 (10.1) | 14 (8.3) | 0.58 |
| Week 26 | 17 (10.2) | 16 (9.5) | 0.86 |
| Week 52 | 16 (10.1) | 15 (9.3) | 0.85 |
| Week 78 | 16 (10.3) | 16 (10.0) | 1.00 |
| Week 104 | 16 (10.3) | 15 (9.7) | 1.00 |
| Antiplatelet drugs |  |  |  |
| Baseline | 15 (8.9) | 10 (5.9) | 0.31 |
| Week 26 | 15 (9.0) | 12 (7.1) | 0.55 |
| Week 52 | 14 (8.8) | 11 (6.8) | 0.54 |
| Week 78 | 14 (9.0) | 11 (6.9) | 0.54 |
| Week 104 | 14 (9.0) | 11 (7.1) | 0.68 |
| Anticoagulants |  |  |  |
| Baseline | 2 (1.2) | 4 (2.4) | 0.68 |
| Week 26 | 2 (1.2) | 4 (2.4) | 0.68 |
| Week 52 | 2 (1.3) | 4 (2.5) | 0.68 |
| Week 78 | 2 (1.3) | 5 (3.1) | 0.45 |
| Week 104 | 2 (1.3) | 4 (2.6) | 0.68 |

Data are presented as number (%) of patients. The two treatment groups were compared by Fisher’s exact test.

**Additional file 1: Table S4. The changes of GSM-CCA on the basis of tertiles of changes in mean-IMT during observation period**

|  | The changes in mean-IMT during observation period | | | P value between groups | | |
| --- | --- | --- | --- | --- | --- | --- |
|  | The 1^st^ tertile | The 2^nd^ tertile | The 3^rd^ tertile | 1^st^ vs. 2^nd^ | 1^st^ vs. 3^rd^ | 2^nd^ vs. 3^rd^ |
| Whole subjects | 0.40±11.15 (n=102) | -0.47±11.83 (n=105) | -1.10±11.64 (n=92) | 0.59 | 0.36 | 0.71 |
| Tofogliflozin group | 0.36±11.14 (n=52) | -0.55±10.72 (n=51) | -2.11±11.73 (n=48) | 0.93 | 0.44 | 0.49 |
| Conventional group | 1.25±10.96 (n=53) | -1.45±13.29 (n=48) | 0.90±11.45 (n=47) | 0.27 | 0.88 | 0.36 |

Data are presented as the mean ± SD unless stated otherwise. The changes of GSM-CCA from baseline to week 104 were compared among the three groups on the basis of tertiles of changes in mean-IMT from baseline to week 104 using Student’s t-test.

CCA, common carotid artery; GSM, Gray-Scale Median; IMT, intima-media thickness.
